# Influence of Annealing Temperature on Weak-Cavity Top-Emission Red Quantum Dot Light Emitting Diode

**DOI:** 10.3390/nano9111639

**Published:** 2019-11-19

**Authors:** Chun-Yu Lee, Ya-Pei Kuo, Peng-Yu Chen, Hsieh-Hsing Lu, Ming Yi Lin

**Affiliations:** 1 AU Optronics Corporation, Hsinchu 30078, Taiwanjoanne.kuo@auo.com (Y.-P.K.); kevinplus2012@gmail.com (P.-Y.C.); d99941004@gmail.com (H.-H.L.); 2 Department of Electrical Engineering, National United University, Miaoli 36003, Taiwan

**Keywords:** QLEDs, top-emission, microcavity

## Abstract

In this report, we show that the annealing temperature in QDs/Mg-doped ZnO film plays a very important role in determining QLEDs performance. Measurements of capacitance and single carrier device reveal that the change of the device efficiency with different annealing temperatures is related to the balance of both electron and hole injection. A comparison of annealing temperatures shows that the best performance is demonstrated with 150 °C-annealing temperature. With the improved charge injection and charge balance, a maximum current efficiency of 24.81 cd/A and external quantum efficiency (EQE) of 20.09% are achievable in our red top-emission QLEDs with weak microcavity structure.

## 1. Introduction

Colloidal semiconductor quantum dots (QDs) have attracted extensive attention as candidates for next-generation display application owing to their unique properties, such as high color purity, narrow full width at half-maximum (FWHM), high photoluminescence quantum yield (PLQY), and simple fabrication process [1,2,3,4,5,6,7,8,9,10,11,12]. A number of approaches for fabrication of cadmium-based QD light-emitting diodes (QLEDs) have been demonstrated, including device structure, material synthesis, and ligand exchange [13,14,15,16,17,18,19,20,21]. However, most of researches are based on bottom-emission structure. For the display applications, top-emission structure is becoming increasingly important. It is because of that top-emission structure has favorable attributes for improving display performance, which include vesting the freedom of pixel and circuit design, increasing the aperture ratio, and lowering power consumption [22]. In this paper, we utilized red top-emission QLED (made by using the all solution-processed device structure) with indium zinc oxide (IZO) as top electrode. To attain excellent device performances and high luminescence efficiency, the device contains emitting layer of CdZnSe/ZnS core/shell QDs with Mg-doped ZnO nanoparticle as the electron transporting layer (ETL) and stacked organic hole transporting layer (HTL). Furthermore, we investigate the impact of thermal annealing on the carrier densities and device performances. It is worth noting that the fabricated QLED reveals an impressive current efficiency of 24.81 cd/A and external quantum efficiency (EQE) of 20.09% with a color co-ordinate (0.687, 0.309), which is the highest performance ever reported in red top-emission QLED structure with IZO electrode.

## 2. Materials and Methods

**Figure 1:**
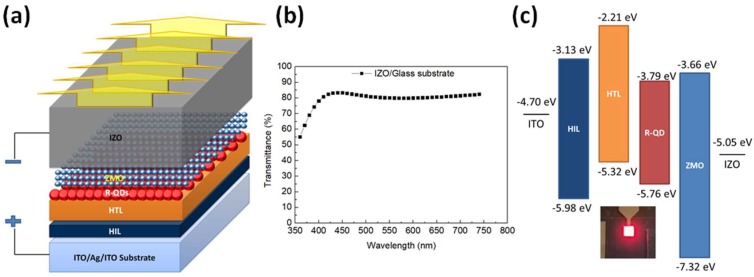
(**a**) Schematic of the device structure. (**b**) Optical transmittance of the IZO/glass substrate. (**c**) Energy band diagram of the red top-emission QLEDs. The inset is a photograph of the working device.

In this report, reference QLED device layer structure consists of a patterned ITO/Ag/ITO glass (Top ITO: 12 nm thick; Ag layer is to reflect light to top electrode), a HIL (20 nm thick), HTL (23 nm thick), QD EML (11 nm thick), ETL (36 nm thick), and IZO cathode (185 nm thick) as schematically shown in Figure 1a. The device is weak microcavity structure, which could effectively eliminate the microcavity effect and enable angular color stability for display applications. To attenuate the optical microcavity effect, a high transparent IZO top contact with a room temperature and low power sputtering process is employed. Figure 1b shows the optical transmittance of the IZO (185 nm) deposited on the glass substrate. The average transmittance in visible range (400–700 nm) is above 80%, which is agreed with the previous reported results [23,24]. It means that the microcavity is weakly formed by the ITO/Ag/ITO anode mirror and IZO cathode. HIL and HTL are the materials for the commercial product of organic light-emitting diodes (OLED). HIL is composed of PFSA (tetrafluoroethylene-perfluoro-3,6-dioxa-4-methyl-7-octenesulphonic acid copolymer), PEDOT:PSS (Poly(3,4-ethylenedioxythiophene)-poly(styrenesulfonate)), and dimethyl sulfoxide. HTL is poly[(9,9-dioctylfluorenyl-2,7-diyl)-co-(4,4′-(N-(4-sec-butylphenyl)diphenylamine)] (TFB) derivative. Red QDs and Zn_0.85_Mg_0.15_O nanoparticles (NPs) are purchased from Mesolight Inc (China). The PLQY of QDs is 70%. Here, Zn_0.85_Mg_0.15_O is Mg-doped ZnO NPs for efficient electron transport and easy injection in the red QDs EML layer.

All the QLED devices are fabricated in the nitrogen-filled glove box. First, we clean the ITO/Ag/ITO substrate by de-ionized water, acetone, and isopropyl alcohol in ultrasonic for 25 min sequentially and then the substrate is treated under UV ozone for 15 min to increase the work function and to improve the adhesion to HIL. Then, HIL is spin-coated at 910 rpm on the ITO/Ag/ITO substrate and baked at 230 °C for 15 min. HTL is spin-coated at 3700 rpm and baked at 230 °C for 30 min. After that, red QDs and Zn_0.85_Mg_0.15_O NPs are deposited layer-by-layer via spin casting on the HTL/HIL/substrate. The red QDs and Zn_0.85_Mg_0.15_O NPs layers are spin-casted both at 3000 rpm. Finally, the transparent IZO top-electrode is deposited by sputtering under a based vacuum of 10^−7^ torr. All devices are encapsulated in a glass-to-glass epoxy sealed package with desiccant. The emitting area is 2 × 2 mm. Figure 1c shows the schematic diagram of fabricated red top-emission QLED structure with energy band/level diagram of each layer. The electronic energy levels are investigated by ultraviolet photoemission spectroscopy (UPS) in a Kratos AXIS ultra-DLD ultrahigh vacuum photoemission spectroscopy system with HeI excitation.

**Figure 2:**
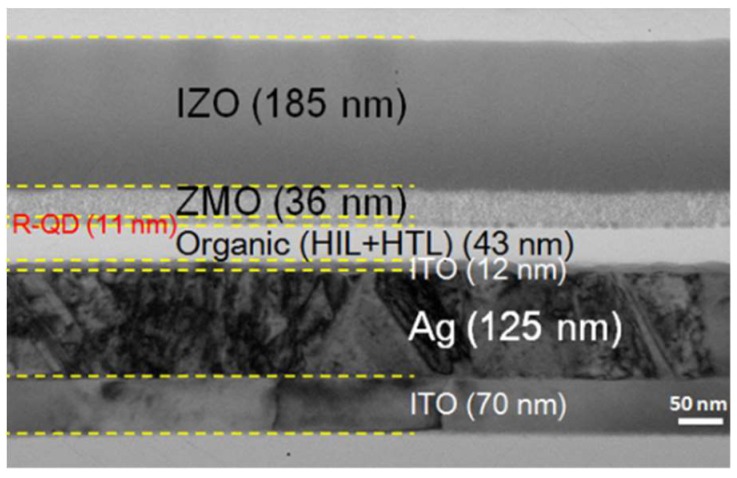
Cross-sectional transmission electron microscopy image of the red top-emission QLEDs.

The corresponding cross-sectional electron transmission microscopy (TEM) image of our QLEDs is shown in Figure 2. The figure clearly shows that a compact QD emitting layer is placed between charge carrier transport layers.

## 3. Results and Discussion

**Figure 3:**
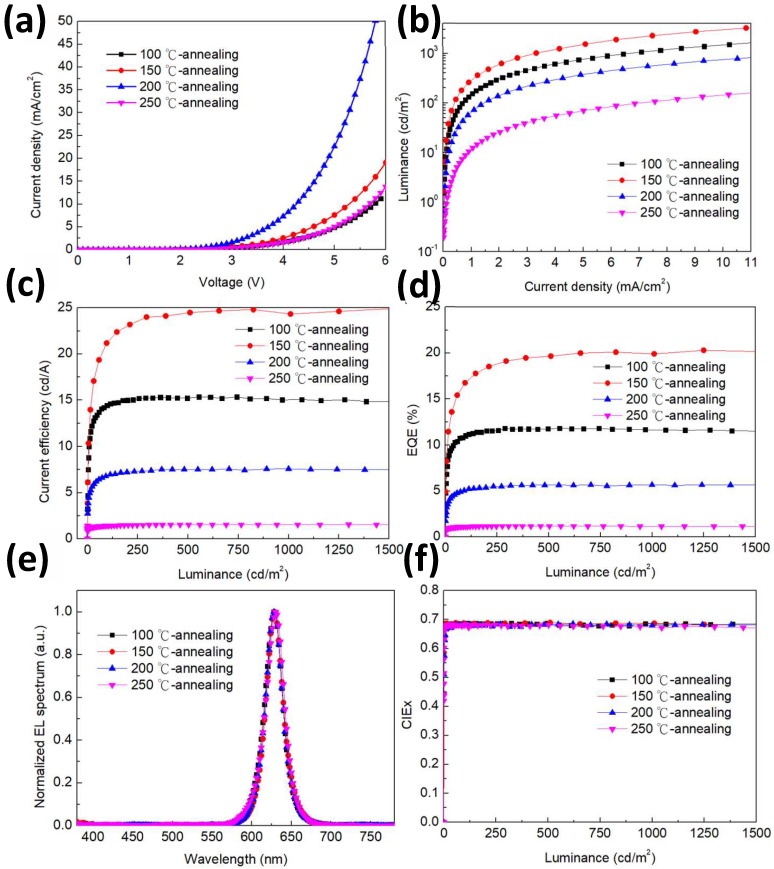
Device performance of the red top-emission QLEDs at different anneal temperatures. (**a**) Current density–voltage (J–V) characteristics. (**b**) Luminance–current density (L–J) characteristics. (**c**) Current efficiency-luminance characteristics. (**d**) EQE-luminance characteristics. (**e**) Normalized EL spectra. (**f**) CIEx-luminance characteristics.

In the experiment, to investigate the thermal annealing on the red top-emission QLEDs, we fabricated four QLEDs at different annealing temperatures of 100, 150, 200, and 200 °C for 10 min respectively after Zn_0.85_Mg_0.15_O NPs layers are deposited on the QDs layers. In addition, the recipe of 100 °C annealing temperatures has been widely used in QLED fabrication [16,17,18]. Figure 3 shows the EL performance of fabricated QLEDs. As J–V characteristics demonstrated in Figure 3a, current density is raised with increasing temperature. It is important to note that with high temperature annealing (>100 °C), the current densities of the QLED devices are higher than that of the device with 100 °C annealing. When the annealing temperature is raised to 150 and 200 °C, the QLED devices have nearly 1.75 and 5.5-fold higher current density than that annealed at 100 °C at the same driving voltage of 6 V, respectively. In contrast, for the device with 250 °C-annealing temperature, the current density is only 1.2-fold higher than that of the device annealed at 100 °C. We attribute this phenomenon to the carrier mobility changed depending on the annealing temperatures. In other words, high annealing temperature (> 100 °C) can improve carrier mobility of QDs/Zn_0.85_Mg_0.15_O O film. Figure 3b–d demonstrate the luminescence–current density (L–J) characteristics and current efficiency-luminescence-EQE properties of these devices. As shown in Figure 3b, the highest luminescence–current density curve is observed at 150 °C annealing temperature. When annealing temperature is increased to 200 °C, the luminescence–current density curve of the QLED device starts to decrease. In particular, as the annealing temperature is further raised to 250 °C, the luminescence significantly decreased. These results suggest that the high temperature degradation of QDs occurred. Figure 3c,d show the current efficiency and EQE of QLEDs, revealing that the best performance is obtained at annealing temperature of 150 °C; it reaches the highest current efficiency of 24.81 cd/A and EQE of 20.09% with a color co-ordinate (0.687, 0.309). Moreover, the devices with annealing temperature of 200 and 250 °C exhibit dramatic decrease in device efficiency. It is clear that the device with 250 °C annealing temperature has the poorest current efficiency of 1.54 cd/A and EQE of 1.15%. The QDs/Zn_0.85_Mg_0.15_O film presents dramatic difference in the device efficiency depending on the annealing temperature. We speculate that the change of the device efficiency is closely related to the balance of both electron and hole injection. The EL spectra of QLEDs are shown in Figure 3e. It is found that the peak wavelength (628 nm) and full-width at half-maximum (FWHM) did not change any more and these devices showed similar CIE properties (see Figure 3f). Owing to Commission Internationale de L’Eclairage (CIE) 1931 color coordinates of (0.687, 0.309) and a narrow FWHM of 26 nm, the color-saturation makes this red top-emission QLED an ideal red array for display application. The detailed EL performance of all red top-emission QLED devices are summarized in Table 1.

**Figure 4:**
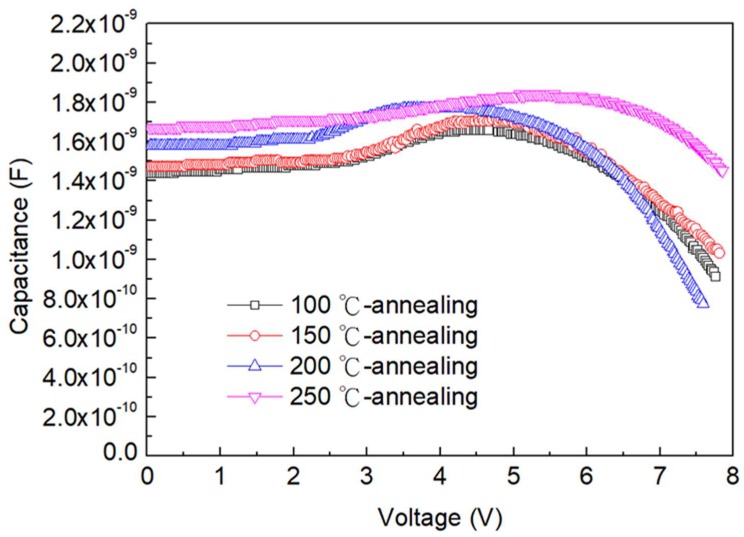
The capacitance–voltage characteristics of the red top-emission QLEDs at different anneal temperatures.

To evaluate the charge balance in our red QLED, we measure the capacitance–voltage (C–V) characteristics of all devices after annealing for different temperatures of 100, 150, 200, and 250 °C. Figure 4 shows the C–V characteristics of the QLEDs. In order to minimize the contribution from traps, the modulating frequency at 100 KHz is used. By doing so, we are able to obtain smooth C–V curves. When a DC bias voltage is applied, a charge-unbalanced device will initiate the fast charge first, resulting in capacitance rise. In a QLED device, the electron mobility is generally higher than the hole mobility. Therefore, the electron injection starts and the injected electrons may accumulate at the interface of QD/HTL. The capacitance of the QLED devices with 100 °C annealing temperature rises at 2.2 V, which means that the HTL is the only layer remaining depleted and other layers have reached flat band and charge injection. At a DC bias voltage of around 4.6 V, the capacitance starts to decrease sharply. It indicates that the efficient radiative recombination of electrons and holes occurred, which is corresponding to the relaxation of accumulated carriers. For the device with 150 °C annealing temperature, the transition voltage corresponding to peak capacitance is unchanged and the peak capacitance is increased to 1.73 nF. These results represent that the charge injection is enhanced. This phenomenon means that an increase of capacitance resulted from efficient injection of both electrons and holes. When annealing temperature is increased to 200 °C, the transition voltage decreases to 1.5 V and peak capacitance increases to 1.79 nF. The decreasing voltage represents that the injection of slow carrier (holes) is enhanced. Moreover, the significantly increased peak capacitance implies that the charge accumulation is enlarged, leading to a more serious charge unbalance. In this case, we can conclude that the carrier increases in electron is much higher than that in holes. For the device further annealing at 250 °C, the transition voltage shifts to 2.45 V and the capacitance peaks at 1.81 nF. The big shift of transition voltage is caused by the degradation of slow charge (holes) injection. Furthermore, the increased capacitance confirms that the electron injection is more efficient than hole injection, and the charge accumulation is highly pronounced.

**Figure 5:**
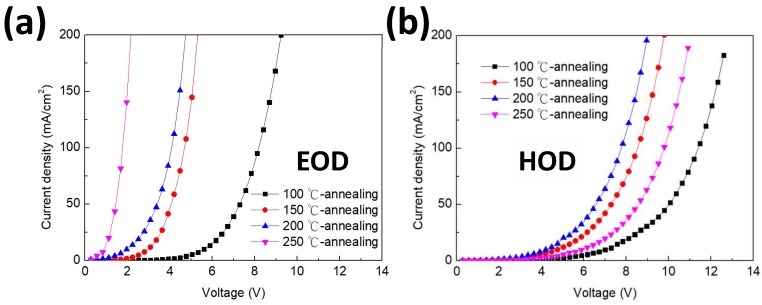
Current density–voltage (J–V) characteristics of (**a**) electron-only devices and (**b**) hole-only devices at different anneal temperatures.

In order to further verify that the increased capacitance is related to the change of the charge injections between electrons and holes by thermal annealing, we prepared the single carrier devices of electron-only devices and hole-only devices and investigated their injection characteristics as described in Figure 5. The electron and hole currents are measured using the devices consisting of ITO/Zn_0.85_Mg_0.15_O/QDs/Liq/Al (EOD) and ITO/HIL/HTL/QDs/HATCN/Al (HOD) with different annealing temperatures of 100, 150, 200, and 200 °C. We also calculated the mobility values from SCLC (space charge limited current) measurements. The current density versus voltage (or electric field) relationship in SCLC region can be expressed by the following equation:

$$J=\frac{9}{8}\varepsilon \varepsilon_{0}\frac{E^{2}}{d}\mu_{0}exp\left(\beta \sqrt{E}\right),$$

where *μ* is mobility of the current carrier, *μ*_0_ is zero-field mobility, *ε* is relative permittivity, *ε*_0_ is vacuum permittivity (8.854 × 10^−14^ F/cm), and *β* is Poole–Frenkel factor. The mobility values are summarized in Table 2. For the electron only device, the electron injection increases in a large range with the increase in annealing temperature. It is likely due to the improvement of Zn_0.85_Mg_0.15_O conductivity that resulted from reduced ZnO defect densities. For the hole only device, when the annealing temperature is raised from 100 to 200 °C, the hole current is also increased with the increase in annealing temperature. It can be seen that with 150 °C annealing temperature, holes in the current shows the comparable increase with electrons current. Hence, the carrier accumulation behavior is similar as that of the device with 100 °C annealing temperature. This indicates that the improvement in holes and electrons current simultaneously may result in enhanced device efficiency. However, with 200 °C annealing temperature the increased range in the holes current of HOD is much less than the electrons current of EOD. Therefore, the carrier accumulation is enhanced and resulted in the low device efficiency. For the HOD with 250 °C annealing temperature, the hole current shows a notable decrease compared with the devices with 150 and 200 °C annealing temperature. The great decreasing hole current indicates that the unbalanced charge is further increased. As a consequence, the device presents the worst EL performance. These measurements of single carrier current also are in agreement with the mobility calculations. As shown in Table 2, μ_e_/μ_h_ shows the minimum value of 12.2 at 150 °C annealing temperature. It indicates that optimization of the mobility is critical to achieving charge balance in QLEDs for high device performance. The above results are consistent with the measurements of EL and C–V characteristics in Figure 3 and Figure 4. Consequently, we believe that an applicable annealing temperature is important to achieving charge balance in QLED which is essential for high performance devices.

## 4. Conclusions

In conclusion, a facile but effective method is proposed to improve the performance of quantum dot light-emitting diode (QLED) by tuning the charge injection between holes and electrons. It is found that the charge injection and device performance are dependent on the annealing temperature used in fabricating the QLED devices. By using an appropriate annealing temperature of 150 °C, we achieved a very high current efficiency of 24.81 cd/A in our red top-emission QLEDs with weak microcavity structure. The investigations on the capacitance–voltage characteristics and the single carrier measurements confirm that much improved electroluminescent efficiency originates from the significant enhancement of charge injection and charge balance.

## Figures and Tables

**Table 1:** Summaries of 1931 CIE (x, y) chromaticity coordinates, electroluminescence emission peak wavelength λ_max_, FWHM, turn on voltage V_T_, current efficiency η_A_, and external quantum efficiency η_EQE_ of the red top-emission QLEDs at different anneal temperatures. Turn on voltage is measured at 1 cd/m^2^.

Weak-Cavity Top-Emission R-QLED	x	y	Peak	FWHM	V_T_	CE (η_A_)	EQE (η_EQE_)
	-	-	nm	nm	V [1 cd/m^2^]	cd/A	%
**100 °C-annealing**	0.684	0.311	628	26	1.9	15.1	11.6
**150 °C-annealing**	0.687	0.307	628	26	1.9	24.8	20.1
**200 °C-annealing**	0.680	0.313	628	25	1.8	7.6	5.7
**250 °C-annealing**	0.671	0.313	628	27	2.6	1.5	1.2

**Table 2:** Electron and hole mobility for single carrier devices at different anneal temperatures.

Unit: cm^2^ V^−1^ s^−1^	100 °C-annealing	150 °C-annealing	200 °C-annealing	250 °C-annealing
**Electron Mobility (μ_e_)**	2.1 × 10^−7^	1.2 × 10^−6^	2.5 × 10^−6^	9.4 × 10^−6^
**Hole Mobility (μ_h_)**	4.2 × 10^−9^	9.8 × 10^−8^	1.2 × 10^−7^	4.2 × 10^−9^
**μ_e_/μ_h_**	50.0	12.2	20.8	2238.1
